# Space division multiplexing in standard multi-mode optical fibers based on speckle pattern classification

**DOI:** 10.1038/s41598-019-53530-6

**Published:** 2019-11-26

**Authors:** Jaël Pauwels, Guy Van der Sande, Guy Verschaffelt

**Affiliations:** Applied Physics research group (APHY), Vrije Universiteit Brussel, Pleinlaan 2, B-1050 Brussels, Belgium

**Keywords:** Fibre optics and optical communications, Imaging and sensing, Photonic devices

## Abstract

In optical communications the transmission bandwidth of single mode optical fibers is almost fully exploited. To further increase the capacity of a telecommunication link, multiplexing techniques can be applied across 5 physical dimensions: amplitude, quadrature, polarization, frequency and space, with all but the latter being nearly exhausted. We experimentally demonstrate the feasibility of an original space division multiplexing technique based on the classification of speckle patterns measured at the fiber’s output. By coupling multiple optical signals into a standard multimode optical fiber, speckle patterns arise at the fiber’s end facet. This is due to quasi-random interference between the excited modes of propagation. We show how these patterns depend on the parameters of the optical signal beams and the fiber length. Classification of the speckle patterns allows the detection of the independent signals: we can detect the state (i.e. *on* or *off*  ) of different beams that are multiplexed in the fiber. Our results show that the proposed space division multiplexing on standard multimode fibers is robust to mode-mixing and polarization scrambling effects.

## Introduction

The incessant demand of optical channels with increasing data rates continues to inspire new developments, as it has for many years^1–4^. A single optical fiber can carry many signals in parallel. Developments such as low-noise and ultra-broadband fiber amplification^5^ and spectrally efficient coding schemes in combination with coherent detection^6^ aim to improve the data rate of each individual channel, whereas thechniques such as time, polarisation and wavelength division multiplexing seek to increase the total number of channels^7–9^. These approaches are typically implemented on single mode fibers. Another way to increase the number of parallel channels is space division multiplexing (SDM)^10–15^. This technique requires a multicore and/or multimode fiber (MCF/MMF) and utilizes the transverse spatial extent of the fiber to create parallel data channels. MMFs have larger cores and can easily support upwards of 100 transverse modes which can be used as data channels. However, this type of fibre is not suited for long-haul telecommunication because of unwanted mode scrambling and intermodal dispersion^3^. The adverse effects of mode scrambling can be somewhat compensated by applying multiple-inputs multiple-outputs techniques^16–18^. The strong temporal broadenening of the transmitted signal limits the maximum data rate. Advancements have been made using few-mode fibers (FMF) which only support a small number of modes^19^. The main disadvantage lies in the difficulty of addressing the individual channels. Coupling light into or extracting light from a particular mode is not straightforward. Alternatively, multiple SDM channels are supported by MCFs in which multiple cores are embedded in the same cladding of a single fiber, with each core supporting 1 mode or channel^20^. Sufficient separation between the fiber cores is required to prevent channel crosstalk. These specialty fibers succesfully mitigate the negative effects of intermodal dispersion but lose compatibility with existing telecom infrastructure. To reach even higher datarates, the two techniques can be combined^19^. When multiple few-mode cores exist within the same fibre, the total number of channels equals the number of cores multiplied by the number of modes or channels per core.

**Figure 1:**
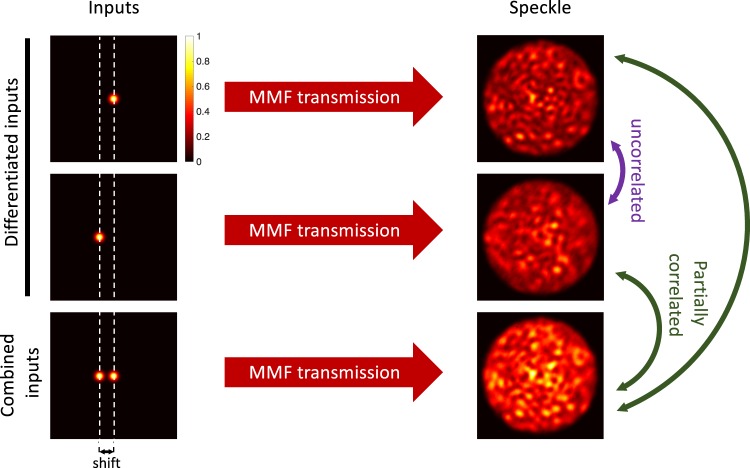
Schematic of speckle-based space division multiplexing using an MMF. At the transmitter side, multiple optical inputs are injected at different positions on the fiber core. At the receiver side, speckle patterns emerge as a result of interference between the fiber’s propagation modes. The inputs are positioned such that each input generates a different speckle pattern. The receiver can identify any combination of inputs through classification of the resulting speckle pattern.

In this work we propose and investigate a new SDM technique that can be used with any type of MMF in a way that exploits propagation mode scrambling. This seemingly random, yet deterministic scrambling transforms a coherent optical input on the transmitter’s end of the MMF into a speckle pattern at the receiver’s end^21,22^. Here, we propose a robust SDM approach based on the classification of these speckle patterns. In Fig. 1 we illustrate our approach (using a standard 62.5 core gradient index MMF) which relies on two key concepts. Firstly, we ensure that individual optical inputs to the MMF at the transmitter side generate uncorrelated speckle patterns at the receiver side. This is achieved with a relative shift of the position of the optical inputs on the fiber’s entrance facet. Secondly, each of these speckle patterns is partially correlated to a new speckle pattern which arises when multiple inputs are injected in the MMF simultaneously. This means that the information carried by each of the inputs is still present in the seemingly random speckle patterns as observed by the receiver.

In the next sections, we test the conditions under which the speckle patterns of different sources become uncorrelated. We also propose and investigate two methods to extract the transmitted data. The first method is used to show that the speckle patterns contain sufficient information, whereas the second method extracts the data in a more efficient way with minimal oversampling of the output intensity pattern. In the discussion section, we analyze the performance limits of our approach.

## Results

### Speckle in MMFs

In a first part, we investigate how the speckle pattern at the fiber’s end facet depends on the input beam. More particularly, we characterize the influence of the source’s incident position, wavelength and polarization. This will allow us to quantify the minimal required parameter variation between different optical inputs to ensure that they generate uncorrelated speckle patterns. In our experiments, we use a standard gradient index MMF because it support many speckle spots. This is important during later stages of our investigations, when we consider the correlations between different speckle patterns. By choosing how much of these spots are used by the processing scheme, such a fiber allows us to investigate how many speckle spots are needed for successful signal recovery.

**Figure 2:**
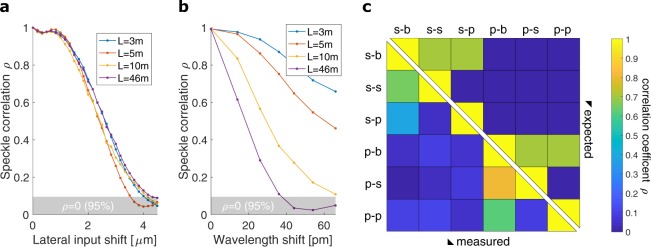
Cross-correlation between speckle patterns generated at (**a**) different input positions on the fiber core (**b**) different wavelengths, for MMF lengths ranging from 3 to 46. The top of the gray bands represents a one-sided confidence bound used to determine if patterns can be considered uncorrelated (see Supplementary Material). In **c** a set of speckle patterns is each labeled by two symbols (e.g. *s*-*b*), the first representing one of two orthogonal polarizations of the input (*s* or *p* injected into the fiber) and the second representing a polarization filter (again *s* or *p*) or the lack thereof (labeled *b*) behind the fiber. For each pair of speckle patterns (or labels) the color-coded correlation above the diagonal represents the expected correlation, whereas the value displayed below the diagonal corresponds to the measured correlation value.

A first parameter that influences the speckle pattern is the position of the incident beam on the fiber’s front facet. This position determines the coupling to the MMF’s propagation modes. Since the speckle pattern at the fiber’s end facet is caused by interference between the excited (and mixed) modes, it will change with the incident spot position. By quantifying the change in the speckle pattern we can determine the minimal separation required between any two signal beams on the fiber’s front facet to ensure they produce uncorrelated speckle patterns. To this end, we record and store a reference pattern on the fiber’s end facet, generated by a beam incident at an arbitrary reference position on the fiber’s front facet. We then compute the correlation between this reference pattern and the patterns generated by beams incident at different positions. Figure 2(a) shows the pattern cross-correlation against the relative shift in incidence position. For all fiber lengths ranging from 3 to 46 a lateral shift of 4 is sufficient to decorrelate the generated speckle patterns. We also observe that the choice of the reference position does not affect these results.

Another parameter which affects the speckle pattern is the source’s wavelength. It is known that changing the wavelength of the light injected in the MMF will alter the modal composition of the injected field and the excited modes will propagate with altered delays^3^. This causes a change in the produced speckle pattern at the fiber’s end facet^23^. To track this change, we first store a reference speckle pattern generated at the source’s nominal operational wavelength. Then we vary the source’s wavelength and store speckle patterns generated at different wavelengths. To quantify the change of the pattern, we calculate the cross-correlation between the reference pattern and each of the successively recorded patterns. The resulting correlation coefficients are plotted against the relative wavelength shift in Fig. 2(b). We find that the correlation between speckle patterns decreases with increasing wavelength difference. To determine which speckle patterns can be considered uncorrelated, a 95% confidence bound (gray band) is constructed (see Supplementary Material). A wavelength shift of 40 is sufficient to remove the pattern cross-correlation in a 46 long MMF link. Larger wavelength shifts are required to decorrelate patterns generated by shorter fiber lengths. Note that the shape of the curve in Fig. 2(b) would be the same if a different reference wavelength was chosen.

A third parameter affecting the speckle pattern is the source’s polarization. Light can be injected in the fiber in two orthogonal polarization modes, here labeled *s* and *p*. The MMF completely scrambles the injected polarization such that the light exiting the fiber always has both *s* and *p* components. The receiver can detect both components (a situation labeled *b*) or choose to filter out a single component. We recorded speckle patterns for different combinations of input polarization (*s* or *p*) and output polarization (*s*, *p*, or *b*). For example, when the transmitter sends *s*-polarized light and the receiver only measures the *p* polarization, the recorded pattern is labeld *s*-*p*. Figure 2(c) shows the cross-correlation coefficients between the recorded patterns. At the intersection between two labels, a colored cell represents the expected (above diagonal cut) or measured (below diagonal cut) cross-correlation. As expected, we find that *s* and *p* polarized speckle patterns are always uncorrelated, and that they are each partially correlated to the unpolarized speckle patterns (e.g. compare. *s*-*s*, *s*-*p* and *s*-*b*). Speckle patterns generated by orthogonal input polarizations are also uncorrelated, regardless of their own polarization state (e.g. compare *s*-*b* and *p*-*b*). Thus, the input polarization can also be used to decorrelate the output speckle pattern.

Beyond these controlled parameters, uncontrolled external perturbations (e.g. fiber displacements) can affect the speckle patterns. As a result, the correlations between speckle patterns generated by individual input sources and/or sets of input sources can be expected to change over time. Evidently, any processing of the speckle patterns which relies on these correlations, needs to take the temporal stability of the system into account to guarantee successful long-term operation. In our experimental setup, stability could be maintained for more than ten minutes. We find that periodic recalibration of the speckle pattern processing scheme can be performed quickly, thus overcoming any temporal stability issues with very little overhead to the transmitted signals. Beyond this approach, (e.g. if the correlations were to change quicker) continuously adaptive processing schemes can be envisioned, based on online learning techniques^24^.

### 4-channel SDM: correlation classifier

**Figure 3:**
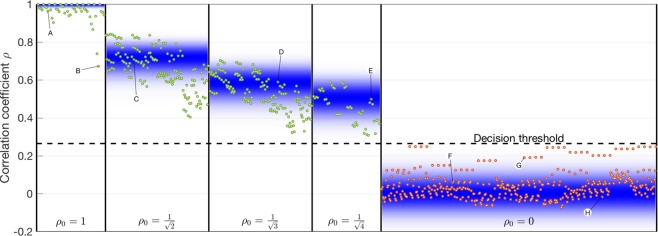
Cross-correlation coefficient *ρ* between reference speckle patterns (generated by individual inputs) and speckle patterns generated by arbitrary combinations of inputs. These results are grouped according to their expected outcome: ${\rho }_{0}=1/\sqrt{n}$ when *n* inputs are active and the resulting speckle patterns is compared with a reference pattern generated by one of those *n* inputs (green markers), and *ρ*_0_ = 0 when compared with any other reference pattern (red markers). The density of the blue bands represents the expected statistical spread of these results, based on the number of available speckle spots for calculating the pattern cross-correlations (see Supplementary Material). Details of the highlighted markers are given in Table 1. The dashed horizontal line represents the decision threshold used to recover the input states: *s*_*i*_ = 1 for cross-correlations above threshold, *s*_*i*_ = 0 below threshold.

Now that we have quantified the influence of the source’s parameters on the generated speckle patterns, we proceed by injecting multiple beams into the MMF simultaneously. In order to test the feasibility of our approach, we use a limited amount of sources: we use 4 optical inputs which are spatially separated on the fiber’s entrance facet. Their relative locations are chosen such that each input produces a different speckle pattern. At each moment in time, the 4-bit signal state is labeled {*s*_1_*s*_2_*s*_3_*s*_4_}, where *s*_*i*_ = 0 or 1 if the corresponding input beam is *off* or *on*. The goal is now to recover each of the 4 laser states *s*_*i*_ by classifying the speckle patterns that appear at the MMF output. In a first readout scheme, the individual signal states are recovered by calculating the cross-correlation between the speckle pattern generated by signal state {*s*_1_*s*_2_*s*_3_*s*_4_} and the pre-recorded reference patterns generated by the individual signal beams, i.e. states {1000}, {0100}, {0010} and {0001}. More particularly, the reference pattern generated by state {1000} is used for the recovery of *s*_1_, {0100} for *s*_2_, and so forth. A unitary correlation coefficient is expected between a reference pattern and a speckle pattern corresponding with the same state that generated the reference pattern. As an example, a speckle pattern corresponding with state {0100} should be fully correlated with the second reference pattern. A partial correlation of $\mathrm{1/}\sqrt{N}$ is expected between a reference pattern and a speckle pattern generated by *n* inputs simultaneously (see Supplementary Material). This is only true if the reference laser under consideration is one of those inputs. For example, the speckle pattern genered by state {0101} should be partially correlated (with an expected of cross-correlation $\mathrm{1/}\sqrt{2}$) only to the reference patterns generated by states {0100} and {0001}. All other cross-correlations (i.e. with the reference patterns {1000} and {0010}) are expected to be zero. A statistical spread on the calculated correlation coefficients is expected due to the finite number of speckle spots in the recorded images (see Supplementary Material) and measurement noise. We performed an SDM experiment with a sequence of all 16 possible 4-bit states {*s*_1_*s*_2_*s*_3_*s*_4_} and repeated this sequence 4 times. Each generated speckle patterns is then compared with each of the 4 reference patterns. This entire measurement is repeated 3 times in order to have sufficient data, leading to a total of 768 calculated correlation coefficients. In Fig. 3 we show these cross-correlations, grouped according to their expected value ($\mathrm{1/}\sqrt{n}$ or 0). Green markers correspond with cross-correlations which are expected to be non-zero. In these cases the signal state to be recovered equals *s*_*i*_ = 1. For all red markers, the expected correlation is zero, as the corresponding signal state equals *s*_*i*_ = 0. Details for the highlighted markers are found in Table 1. The density of the blue bands in the background of Fig. 3 represent the expected statistics, based on the number of active signal states and the number of speckle spots in the images (see Supplementary Material). We find that the measured cross-correlations correspond well with the theoretical expectations. More importantly, the ensemble of green markers is linearly separable from the red markers. This means that for a speckle pattern generated by any state {*s*_1_*s*_2_*s*_3_*s*_4_}, each of the individual signal states can be recovered without error. More specifically, this is achieved by comparing the cross-correlation coefficients to a scalar decision threshold. A good threshold value lies halfway between 0 and the lowest non-zero correlation (here $\mathrm{1/}\sqrt{4}$) at 0.25 (a more refinemed estimate is discussed in Methods). This threshold is visualized as a horizontal dashed line in Fig. 3. The receiver should map *s*_*i*_ to 1 if the correlation exceeds the threshold, and map *s*_*i*_ to 0 below threshold.

**Table 1:** For the highlighted markers in Fig. 3 we show the input states corresponding with the recorded speckle pattern, the reference pattern to which we are comparing it, and both the expected and measured cross-correlation between those two speckle patterns.

Label	Sequence	Reference	*ρ*_0_ expected	*ρ* measured
A	{1000}	{1000}	1	0.96
B	{0001}	{0001}	1	0.67
C	{1100}	{0100}	$1/\sqrt{2}\approx 0.71$	0.71
D	{1011}	{0010}	$1/\sqrt{3}\approx 0.58$	0.59
E	{1111}	{0001}	$1/\sqrt{4}=0.50$	0.50
F	{1010}	{0100}	0	0.06
G	{0000}	{0010}	0	0.19
H	{0100}	{0001}	0	−0.02

### 4-channel SDM: linear classifier

**Figure 4:**
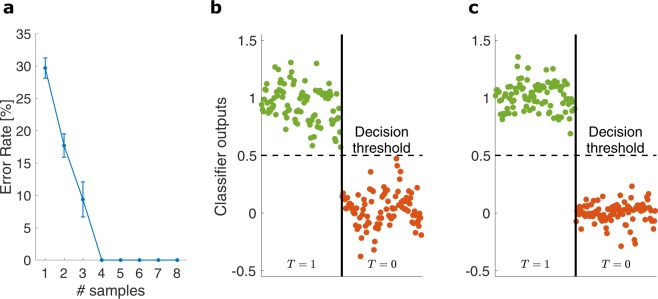
(**a**) Plots the total classifier error rate against the number of speckle intensities sampled from each of the recorded images. The classifiers’ outputs on the validation set are shown when (**b**) 4 samples (**c**) 6 samples are used to classify the speckle patterns. These outputs are grouped and color-coded according to the target output (either *T* = 1 or *T* = 0). The dashed horizontal line represents the decision threshold used to recover the input states: *s*_*i*_ = 1 for classifier outputs above threshold, *s*_*i*_ = 0 below threshold.

Here, we explain an alternative readout scheme and validate it on the same experimental data as in the previous section. In this scheme, the signal states of the 4 independent SDM channels through the MMF link are recovered using linear classifiers^25^. This allows for a more efficient extraction of the input data, i.e. using a small number of measurements. To show the strength of this classification method, we sparsely sampled the speckle patterns by only retaining small subsets of pixels from the recorded images. The classifier outputs are constructed as weighted sums of input features, here the sampled speckle intensities: we sample the intensity of the speckle pattern at different locations accross the output facet, such that 2 adjacent samples are separated by a distance larger than the size of 1 speckle spot. For each SDM channel, a set of readout weights needs to be optimized such that the classifier’s output can reconstruct the corresponding signal state. To train the classifiers, the set of recorded speckle patterns is first divided into a larger training set and a smaller validation set. The former is used to train the readout weights and the latter to evaluate the classifier’s performance (see Methods). An error rate is calculated by dividing the number of misclassified bits by the total number of bits sent. In Fig. 4(a) we plot the total error rate (cumulative over all channels) against the number of speckle intensities sampled from the recorded images. The errorbars correspond with performance fluctuations over iterations with different sampling locations. We find that at least 4 intensity samples are needed to obtain error-free recovery of all 4 signal states. This corresponds to just one sample per SDM channel, i.e. there is no oversampling of the output pattern. In Fig. 4(b) we show the classifier outputs when 4 intensity samples are used. Since the classifiers are trained to reproduce the signal states, these results are grouped according to the target outputs, either *T* = 1 (green markers) or *T* = 0 (red markers). We find that the classifier outputs are linearly separable, which means that the signal states can be recovered without error. This is achieved by comparing the classifier outputs to a decision threshold halfway between the target outputs at 0.5. Outputs above this threshold correspond with *s*_*i*_ = 1, and *s*_*i*_ = 0 below threshold. Further, in Fig. 4(c) we show the classifier outputs when 6 speckle intensity samples are used. It is clear that this small overhead in the number of samples significantly increases the linear separability between the two measurement groups. In this case, the classifiers are robust to higher levels of noise.

## Discussion

We have succesfully demonstrated the feasibility of a novel SDM scheme with a 4-channel demonstrator. At the transmitter side, multiple optical inputs are injected into a gradient index MMF. The mode scrambling during propagation in the fiber is exploited as it leads to the emergence of speckle patterns at the receiver’s end of the fiber. Through control of the position of the optical inputs we can ensure that each input leads to a different speckle pattern. This differentiation is critical, as the recovery of the injected data is achieved through the classification of these speckle patterns. We have proposed and tested two such classification schemes, were we strived to keep the detection and post-processing complexity minimal.

We have found that speckle patterns generated at different wavelengths are uncorrelated after propagation through the MMF (if the wavelength difference is sufficient). The same holds for speckle patterns generated by orthogonal polarizations. Note that the orthogonality of speckle patterns is precisely the requirement for speckle patterns generated by each of the space-multiplexed inputs. This means that the receiver’s speckle classifier could just as well process the patterns generated by different wavelengths or polarizations as independent data channels. This becomes of interest when the speckle classifier is potent enough to classify more patterns than there are SDM channels. In such a scenario, multiple wavelengths or polarizations per SDM channel could be processed by the same classifier. This way, less wavelength or polarization dependent filters are needed to implement wavelength or polarization division multiplexing.

A first speckle classification scheme was based on the linear separability of the cross-correlations between the emerging speckle patterns and a set of pre-recorded reference patterns. The training of these correlation-based classifiers is extremely easy, as it simply consists of recording one reference pattern per SDM channel. This classification method relies on full-view recordings of the speckle patterns. The camera used in these experiments suited our investigations but is too slow to demonstrate the high-speed potential of this novel SDM approach. Therefore, we also investigated an alternative classification scheme which can overcome this issue. In this scheme, a set of linear classifiers was trained to reconstruct the injected data through linear combinations of sampled speckle intensities. Training of these classifiers requires processing speckle patterns generated by multiple combinations of input signal states. The sampling of the speckle patterns can in practice be achieved by a detector array. Since the detectors in such an array only need to sample the local speckle intensities (rather than full-view imaging), commercially available fast detectors can be used. The goal of this paper, however, is to test the feasibility of the proposed SDM concept. The high-speed testing of the data extraction by using fast detectors is beyond the scope of this paper. Similarly, testing longer fiber distances and online retraining of the classifier in order to compensate for drifts in the speckle patterns will be the subject of future work.

One limitation of the proposed SDM concept lies in the number of channels an MMF can support. To quantify this, we consider the minimal required spatial separation of optical inputs on the fiber’s entrance facet. Each input can be said to occupy the area of a sphere with a diameter equal to this minimal separation, approximately 4 in our experiments. Close-packing of these equal spheres yields a maximum packing density *η* of just over 90%. With *A*_*input*_ the area occupied by each input, and *A*_*fiber*_ the area of of the MMF core, we can thus calculate the maximal number of inputs *N* which could operate as parallel SDM channels. We find *N* ≤ *ηA*_*fiber*_/*A*_*input*_ and for an MMF with a core diameter of 62.5 this results in *N* ≤ 220. In such a scenario however, the number of channels supported by this SDM approach is more likely limited by the potence of the receiver’s speckle classification method. The correlation-based classifier has to separate non-zero cross-correlations as low as $\mathrm{1/}\sqrt{N}$ from zero-mean cross-correlations. This becomes harder for larger *N*. A partial solution would be to reduce the statistical noise which distorts these correlation coefficients. This can be achieved by increasing the number of speckle spots (currently $\sim 300$)^21^, e.g. by using an MMF with a larger core. On the other hand, the classification results obtained with the linear classifiers suggest that the number of speckle intensity samples should only exceed the number of SDM channels by a small margin in order to obtain robust operation. Therefore, also the number of speckle spots across the fiber end facet only needs to exceed the number of SDM channels by a small margin. As a rule of thumb, when fewer SDM channels are required, then also fewer fiber modes are needed to produce the required amount of speckle spots. So in this classification scheme, the use of an FMF (with smaller core size) is actually favourable compared to an MMF. In general, the classification becomes harder for large *N*.

The main advantage of using MMFs rather than SMFs, is obviously the possibility of implementing SDM. As with any multiplexing scheme, it has the potential of boosting the total channel capacity proportional with the number of parallel channels. However, a drawback of using MMFs for long-haul datatransmission is intermodal dispersion. This limits the maximal modulation rate of the input signal, and thus limits the channel capacity. Combining these considerations, our SDM method would actually benefit from switching to FMFs, where intermodal dispersion can (at least partially) be mitigated. In an ideal case then, the number of fiber modes is matched to the number of parallel SDM channels one wishes to implement. For example, in a scenario where we wish to implement 10 SDM channels, we would switch from a large-core MMF to a suitable FMF with just over 10 modes. We expect that for such FMFs the index profile can be designed in order to significantly reduce intermodal dispersion^26,27^. This way, the penalty to the total channel capacity on account of this dispersion, would not outweigh the boost due to the parallel channels. Compared to other techniques that utilize such FMFs, the proposed SDM approach has the advantage that it does not require the excitation/detection of the individual modes of the FMF, which strongly simplifies the in/out coupling of the light beams in the FMF. Moreover, training of the output classifier is relatively straightforward. In conclusion, the proposed approach can be used to implement SDM both in short distance datacom using MMFs and in long distance systems employing FMFs.

## Methods

### Speckle in MMFs: setup

**Figure 5:**
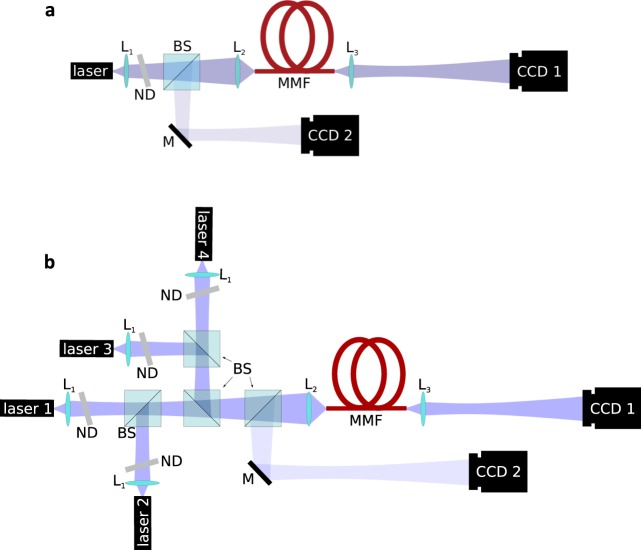
(**a**) Schematic representation of the fiber speckle measurement setup with HL6358MG laser diode operating at 639, beam splitter *BS*, neutral density filter *ND*, multimode fiber (*MMF*) sections with total length ranging from 3 to 46, lenses *L*_1,2,3_ with focal lengths *f*_1_ = 11 mm, *f*_2_ = 8 mm and *f*_3_ = 9 mm, mirror *M* and sensors *CCD*_1_ and *CCD*_2_. (**b**) 4-channel SDM demonstrator setup obtained by expanding setup **(a)** with 3 additional lasers, lenses, filters and beamsplitter, to allow for multiple optical inputs to the MMF. *CCD*_2_ monitors input positions on the fiber core at the transmitter side. *CCD*_1_ records the emerging speckle patterns at the receiver side.

The speckle measurement setup is schematically illustrated in Fig. 5(a). A single laser beam is injected into the MMF. The optical source is a Thorlabs HL6358MG laser diode operating around 639. The strongly divergent light emitted by the diode is first collimated using lens *L*_1_ and then focused onto the fiber’s front facet using lens *L*_2_. The placement of a neutral density filter allows us to attenuate reflections that feed back into the laser. To obtain an image of the beam on the fiber’s front facet, a beamsplitter reroutes the light that reflects off the facet towards a CCD sensor (labeled *CCD*2). Using this image, the beam diameter on the fiber’s front facet was determined to be 5, which is small compared to the 62.5 diameter of the fiber core. Commercially available gradient index MMFs are used with lengths varying from 3 to 46. The injected beam excites several modes of the MMF which interfere to form the speckle pattern at the fiber’s end facet. A single lens (*L*_3_) is used to image this pattern on a high resolution Spiricon 12-bit CCD camera, model SP620U (labeled *CCD*1).

Our setup allows to control the wavelength of the optical source by changing the laser diode’s temperature and/or injected current. The laser is always operated in its single-longitudinal mode regime. The polarizations of the injected and measured fields are controlled by placing polarizers in front of and behind the MMF.

### 4-channel SDM: setup

Compared to the speckle characterisation setup explained in the previous section, 3 additional lasers are introducted to obtain the 4 channel SDM demonstrator setup. All 4 laser beams are collimated, attenuated as necessary, and then redirected towards the fiber’s entrance facet using 3 additional beamsplitters. This means that all 4 lasers can now contribute to the speckle pattern which forms on the fiber’s end facet, and which is imaged on *CCD*1 by lens *L*_2_. Again, an image of the fiber’s front facet is captured by *CCD*2 to monitor the incidence positions of all 4 signal beams. In the SDM experiments these incidence positions are used to differentiate the signal beams, which is a neccesary condition to obtain independent SDM channels. In the SDM experiments, the lasers are directly modulated independently with 4 time-encoded binary signals, employing an on-off keying (OOK) scheme. This is why the state (on or off) of the *i*^*th*^ signal is represented by a binary variable *s*_*i*_(*t*) where *t* represents discrete time. Consequently, the 4-bit sequence sent at time *n* is the combination of all signal states, represented as {*s*_1_*s*_2_*s*_3_*s*_4_}(*t*).

### Speckle contrast

The amount of speckle on a uniformly illuminated screen is quantified by the speckle contrast C, defined as the ratio between the standard deviation *σ*_*s*_ of observed intensity fluctuations and the mean intensity *I*_*s*_ on the screen, *C* = *σ*_*s*_/*I*_*s*_. We have to normalize the recorded speckle patterns of the fiber’s end facet in a pre-processing step before calculating the speckle contrast. This additional processing step takes into account the non-uniform beam shape of the light exiting the fiber.

Since the different signal beams are not coherently related to each other in this experiment, the speckle patterns genereted by different signal beams will add up incoherently. This means that the patterns generated by multiple beams have a lower speckle contrast than the patterns generated by any single beam, of which the speckle contrast *C*_1_ is approximately $\mathrm{1/}\sqrt{2}$ due to polarization diversity. When *n* lasers are on simultaneously, the speckle contrast is reduced to ${C}_{1}/\sqrt{n}$. In general, a lower speckle contrast is expected to make the pattern classification task more difficult, as in this context the speckle contrast can be viewed as a signal-to-noise ratio. The drop in speckle contrast could be mitigated by using coherently related beams as optical inputs^22^, e.g. derived from the same laser and modulated individually. In this case, the speckle contrast is not affected by the number of active input beams *n*. However, the cross-correlations between coherently superposed speckle patterns and its constituent patterns will decrease faster, with 1/*n* (see Supplementary Material).

### Correlation-based classifier

For two speckle patterns *A* and *B*, the intensities captured by the *i*^*th*^ pixel in the image are labeled *A*_*i*_ and *B*_*i*_. For images with *N* (real-valued) pixels, the Pearson cross-correlation coefficient *ρ*(*A*, *B*) is defined as

$$\rho (A,B)=\frac{1}{N-1}\mathop{\sum }\limits_{i=1}^{N}\,(\frac{{A}_{i}-{\mu }_{A}}{{\sigma }_{A}})(\frac{{B}_{i}-{\mu }_{B}}{{\sigma }_{B}})$$

with *μ* and *σ* the respective means and standard deviations. It is worth noting that the recorded speckle images are processed before the cross-correlations are calculated. This pre-processing step filters out the slow intensity profile across the fiber’s end facet, i.e. the profile one would observe if the fiber modes would not be able to interfere and generate speckle. This way, only intensity fluctuations due to speckle are considered in these cross-correlations.

For the decision making of the correlation-based classifiers, a good value for the decision threshold can be obtained a posteriori from the sampled correlation coefficients (i.e. after recording all possible sequences). However, based on the PDFs of the sampling correlation coefficients we can calculate the probability of a given threshold value yielding correct classification of all possible sequences. And we can thus a priori estimate the optimal decision threshold by numerically optimizing this likelihood. These calculations yield a value of 0.26 of the decision threshold, which is close to the original estimate of 0.25 (halfway between $\mathrm{1/}\sqrt{4}$ and 0) mentioned in the Results section.

### Linear classifier

The linear classifier operates on a small subset of pixels from the recorded speckle patterns generated by sequences {*s*_1_*s*_2_*s*_3_*s*_4_}. One classifier is constructed for each input signal beam (or each SDM channel) and the goal of such a classifier *i* is to assign all speckle patterns *V* generated by sequences {*s*_1_*s*_2_*s*_3_*s*_4_} to one of two classes: a class for patterns where the corresponding signal beam was active (*s*_*i*_ = 1) at the time of recording and another class for patterns where it was not (*s*_*i*_ = 0). So in essence, the *i*^*th*^ classifier has to reconstruct the *i*^*th*^ signal state *s*_*i*_. In our case, linear classifiers will suffice, since any speckle pattern at the fiber’s end facet is simply a linear combination of some or all reference patterns.

A speckle pattern *X*(*t*) recorded at timestep *t* is a collection of *N* recorded intensity values {*x*_*j*_(*t*)} (1 ≤ *j* ≤ *N* with *N* the total number of pixels in the images). In order to reconstruct signal state *s*_*i*_(*t*) the *i*^*th*^ classifier uses a set of readout weights *w*_*ji*_ (1 ≤ *j* ≤ *N*) to compute a classifier output *z*_*i*_(*t*). To reduce the computational intensity of this operation, only a subset of *N*_*s*_ ($\ll N$) pixels is used and relabeled {*x*_*j*_(*t*)} (1 ≤ *j* ≤ *N*_*s*_) to obtain

$${z}_{i}(t)=\mathop{\sum }\limits_{j=1}^{{N}_{s}}\,{x}_{j}(t){w}_{ji}$$

the *i*^*th*^ signal state is then reconstructed by passing this classifier output through a threshold-based decision function *f* with

$$f(z)=\{\begin{array}{ll}0 & {\rm{if}}\,z < {z}_{th}\\ 1 & {\rm{if}}\,z\ge {z}_{th}\end{array}$$

where *z*_*th*_ is the decision threshold. In matrix formalism, we can combine all classifier outputs for all of the *T* timesteps *t* (1 ≤ *t* ≤ *T*) in our experiment.

$$Z=X{W}_{out}$$

Here *Z* is the *T* × 4 classifier output matrix where the element (*Z*)_*ti*_ is the output of the *i*^*th*^ classifier at timestep *t*, *X* is the *T* × *N*_*s*_ input matrix where the element (*X*)_*tj*_ is the intensity of the *j*^*th*^ pixel in the (subset selected from the) image recorded at the *t*^*th*^ timestep, and *W*_*out*_ is the *N*_*s*_ × 4 matrix of readout weights where the element (*W*_*out*_)_*ji*_ represents the *j*^*th*^ readout weight of the *i*^*th*^ classifier *w*_*ji*_.

To obtain the readout weights *W*_*out*_ we provide the classifiers with the target output for a subset of *T*_1_ (<*T*) recorded images called the training set. The *T* × 4 target output matrix $\hat{Z}$ holds the desired classifier outputs for all recorded images, where the element ${(\hat{Z})}_{ti}$ corresponds with the known *i*^*th*^ signal state at timestep *t*: *s*_*i*_(*t*). For the subset of images that makes up the training set, the input matrix *X*_1_ is limited to the corresponding subset of rows of *X*, and similarly ${\hat{Z}}_{1}$ is constructed from rows of $\hat{Z}$. The classifier readout weights *W*_*out*_ are obtained as

$${W}_{out}={X}_{1}^{\dagger }{\hat{Z}}_{1}$$

where the pseudo-inverse operator ${\bullet }^{\dagger }$ is used to obtain a least squares solution for the error between the classifier outputs and the target outputs

$${W}_{out}=\mathop{{\rm{a}}{\rm{r}}{\rm{g}}{\rm{m}}{\rm{i}}{\rm{n}}}\limits_{W}||{X}_{1}W-{\hat{Z}}_{1}{||}_{2}^{2}$$

to validate the training, we consider the recorded speckle patterns which were not in the training set. These images constitute the test set and contain information the classifiers have not seen before. The corresponding input matrix *X*_2_ is combined with the optimized readout weights *W*_*out*_ to obtain the classifier outputs. The reconstructed signal states are obtained by passing these outputs through the decision function *f*(*z*) with the threshold set to *z*_*th*_ = 0.5, and are compared with the known signal states, collected in ${\hat{Z}}_{2}$. In other words, both ${\hat{Z}}_{2}$ and *f*(*X*_2_*W*_*out*_) contain only 1’s and 0’s corresponding with the known and reconstructed signal states respectively, and can thus be compared element-wise. To quantify performance the error rate is obtained by dividing the number of misclassified input states by the total number of states in the test set.

## Supplementary information

Supplementary Information
