# Supplementary material for: Space division multiplexing in standard multi-mode optical fibers based on speckle pattern classification
Source: Sci Rep. 2019 Nov 26;9:17597. doi: 10.1038/s41598-019-53530-6 (PMC6879645; doi:10.1038/s41598-019-53530-6)
Supplement: Supplementary file 1 — Supplementary Information [file 41598_2019_53530_MOESM1_ESM.pdf]

Space division multiplexing in standard multi-mode optical fibers based on speckle pattern classification

Jaël Pauwels, Guy Van der Sande and Guy Verschaffelt

Supplementary Information

Speckle correlation statistics: inputs not coherently related

When multiple input signals are active, the corresponding speckle pattern is partially correlated to the reference speckle patterns generated by each of the active input signals individually. To predict these non-zero partial correlations, consider the general case with n ($0 \leq n \leq 4$) active signal beams producing overlapping speckle patterns. The speckle intensity patterns generated by individual signal beams can be considered as independent and identically distributed random variables R_i ($i = 1,...,4$) where intensity spots in the captured images represent random samples or realizations of these variables. Due to incoherent superposition, the speckle intensity pattern P generated by any sequence $\{s_1s_2s_3s_4\}$ (with s_i representing binary laser states) can be written as a linear combination of the reference patterns R_i.

$$P = s_1R_1 + s_2R_2 + s_3R_3 + s_4R_4$$

Let us collect the indices of the n active lasers in set J such that

$$J = \{i \in \{1,2,3,4\} \mid s_i = 1\}$$

For example, if $\{s_1s_2s_3s_4\} = \{0101\}$ then J has two elements, namely $J_1 = 2$ and $J_2 = 4$. The expression for the speckle pattern P then becomes

$$P = \sum_{i=1}^{n} R_{J_i}$$

We know that the covariance operator is a bilinear, i.e.

$$\text{Cov}(R_i,P) = \text{Cov}(R_i, \sum_{j=1}^{n} R_{J_j}) = \sum_{j=1}^{n} \text{Cov}(R_i,R_{J_j}),$$

we then find the sample cross-correlation coefficient between reference pattern R_i and speckle pattern P

$$\begin{aligned}\text{Corr}(R_i,P) =& \frac{\text{Cov}(R_i,P)}{\sqrt{\text{Var}(R_i)\,\text{Var}(P)}}\\ =& \frac{\sum_{j=1}^{n}\text{Cov}(R_i,R_{J_j})}{\sqrt{\text{Var}(R_i)\left(\sum_{j=1}^{n}\sum_{k=1}^{N}\text{Cov}(R_{J_j},R_{J_k})\right)}}\end{aligned}$$

Since we made sure the individual signal beams generate uncorrelated patterns, we have $\text{Cov}(R_{J_j},R_{J_k}) = \sigma^2\delta_{J_jJ_k}$ and through the construction of J we have that $\delta_{J_jJ_k} = \delta_{jk}$. When comparing one of the reference patterns R_i with a pattern P generated by n active signal beams, one of which generated the reference pattern R_i, we have $i \in J$. In this case, we find

$$\text{Corr}(R_i,P) = \frac{\sum_{j=1}^{n}\sigma^2\delta_{iJ_j}}{\sqrt{\sigma^2\left(\sum_{j=1}^{n}\sum_{k=1}^{n}\sigma^2\delta_{jk}\right)}} = \frac{\sigma^2}{\sqrt{n\sigma^4}} = \frac{1}{\sqrt{n}} \triangleq \mu_\rho(n)$$

Alternatively, when comparing one of the reference patterns R_i with a pattern P generated by n active signal beams, none of which generated the reference pattern R_i, we have $i \notin J$. In this case, we find $\mu_\rho = \text{Corr}(R_i,P) = 0$. As we combine up to 4 signals ($n \leq 4$), this motivates the division of our measurement results into 5 groups: we expect to find 48 correlation coefficients close to 1, 144 coefficients close to $1/\sqrt{2}$, 144 coefficients close to $1/\sqrt{3}$, 48 coefficients close to $1/\sqrt{4}$ and 384 coefficients close to 0.

We can go a step further and construct the non normal probability density functions (PDF) for these correlations per group. We can do this using the Fisher transformation F, requiring only the expected value of the correlation coefficient and the sample

size, which in this case corresponds to the number of speckle spots m in each pattern. The Fisher transform z is a useful tool in determining the distribution of the sample correlation coefficient ρ between normally distributed bivariate sample pairs with expected correlation μ_ρ. The Fisher variable z is obtained as

$$z = F(\rho) = \text{atanh}(\rho)$$

and by good approximation it follows a normal distribution with mean and variance

$$\mu_z(\mu_\rho, m) = \text{atanh}(\mu_\rho) + \frac{\mu_\rho}{2m}$$
$$\sigma_z^2(\mu_\rho, m) = \frac{1}{m} + \frac{6-\mu_\rho^2}{2m^2}$$

We cannot use these results directly as our sample pairs represent speckle intensities which are not normally distributed. Taking into account the correct distribution of n (> 1) overlapping speckle intensities (assumed equally strong and having m speckle spots), we calculated that the Fisher variable z now has mean and variance

$$\mu_z(n,m) = \text{atanh}(\frac{1}{\sqrt{n}}) - \frac{n-7}{4\sqrt{n}(n-1)m}$$
$$\sigma_z^2(n,m) = \frac{1}{m}\frac{2n+1}{2n-2}$$

We will assume that these characteristics are still sufficient to accurately describe the distribution of z. Since $F^{-1}(z)$ is monotonic we can obtain the non-normal PDF of ρ from the normal PDF of z using

$$p_\rho(\rho) = \left.\frac{p_z(z)}{|\frac{d}{dz}F^{-1}(z)|}\right|_{z=F(\rho)}$$

Since the inverse Fisher transfom is given by $F^{-1}(z) = \tanh(z)$ and since $dF^{-1}(z)/dz = 1 - \tanh^2(z) = 1 - (F^{-1}(z))^2$ this expression can be further simplified. We arrive at the PDF of the sample correlation coefficient ρ as a function of the number of active lasers n and the number of speckle spots m.

$$p_{\rho,n,m}(\rho) = \frac{p_z(\text{atanh}(\rho))}{1-\rho^2} = \frac{1}{(1-\rho^2)\sqrt{2\pi\sigma_z^2(n,m)}} \exp\left(-\frac{(\text{atanh}(\rho) - \mu_z(M,N))^2}{2\sigma_z^2(n,m)}\right)$$

These PDFs are compared with the measurement groups where partial or zero cross-correlations are expected. For the group of results with expected unity correlation, the PDF should in theory be a δ-function at $\rho = 1$. Only measurement noise and temporal drift of the speckle patterns is expected to induce some variance to these measurements.

Speckle correlation statistics: inputs coherently related

When all the signal beams are coherently related, the speckle patterns generated by the individual lasers will interfere coherently at the fiber's end facet. As such, the expected correlation between these combined patterns and the reference patterns scales differently with the number of active lasers n. In the case where the signal beams are not coherently related, we found $\mu_\rho^{incoh}(n) = 1/\sqrt{n}$. We will show that in the coherent case we have $\mu_\rho^{coh}(n) = 1/n$ (assuming all patterns have equal mean intensities).

In this coherent regime we cannot simply work with recorded intensities. Instead we must keep track of the amplitude A and phase θ of the electric field E, or equivalently the real and imaginary part, denoted X and Y respectively, with

$$\begin{cases} \text{Re}\{E\} = X = A\cos\theta \\ \text{Im}\{E\} = Y = A\sin\theta \end{cases}$$

The joint probability density function of the field components X and Y corresponding to a fully-developed speckle pattern is known to be

$$\begin{aligned} p_{X,Y}(X,Y) &= \frac{1}{2\pi\sigma^2}\exp\left(-\frac{X^2+Y^2}{2\sigma^2}\right) \\ &= \frac{1}{\sqrt{2\pi\sigma^2}}\exp\left(-\frac{X^2}{2\sigma^2}\right) \cdot \frac{1}{\sqrt{2\pi\sigma^2}}\exp\left(-\frac{Y^2}{2\sigma^2}\right) \\ &= p_X(X)p_Y(Y) \end{aligned}$$

where σ^2 is related to the mean intensity of the speckle pattern, as can be seen from the corresponding probability density function of the speckle intensity I

$$\begin{aligned} p_I(I) &= \frac{1}{2\sigma^2}\exp\left(-\frac{I}{2\sigma^2}\right) \\ &= \frac{1}{I_0}\exp\left(-\frac{I}{I_0}\right) \end{aligned}$$

with mean intensity $\langle I\rangle = 2\sigma^2 = I_0$. In the incoherent case, we said that speckle spot intensities were considered to be sets of samples X_i of the (then unspecified) speckle intensity distribution. Now, in the coherent case, we will consider the field components at these speckle spots X_i and Y_i to be a set of bivariate samples from $p_{X,Y}(X,Y)$. Note that the field components are uncorrelated since $p_{X,Y}(X,Y)$ can be written as the product of the marginal distributions $p_X(X)$ and $p_Y(Y)$. X and Y are both normally distributed with mean $\langle X\rangle = \langle Y\rangle = 0$ and variance $\mathrm{Var}(X) = \mathrm{Var}(Y) = \sigma^2$. Due to the coherent superposition, the intensity pattern P generated by n coherent beams can now be written in terms of the composing field components X_j and Y_j ($j = 1,...,N$). We now consider the ith correlation classifier which has to detect wether the ith beam is on. This means we are comparing the reference pattern R_i based on X_i and Y_i with a pattern P based on the square of the sum Z_X of all X_j and the square of the sum Z_Y of all Y_j for $j \in J$ with (the same definition as in the previous section)

$$J = \{i \in \{1,2,3,4\} \mid s_i = 1\}$$

such that

$$\begin{cases} R_i = X_i^2 + Y_i^2 \\ P = Z_X^2 + Z_Y^2 = \left(\sum_{j\in J} X_j\right)^2 + \left(\sum_{j\in J} Y_j\right)^2 \end{cases}$$

Note that $\langle Z_X\rangle = \langle Z_Y\rangle = 0$ and $\mathrm{Var}(Z_X) = \mathrm{Var}(Z_Y) = N\sigma^2$. We are establishing the expected cross-correlation coefficient between patterns R_i and P

$$\mathrm{Corr}(R_i,P) = \frac{\mathrm{Cov}(R_i,P)}{\sqrt{\mathrm{Var}(R_i)\,\mathrm{Var}(P)}}$$

Applying the bilinear covariance operator to R_i and P we find

$$\begin{aligned} \mathrm{Cov}(R_i,P) &= \mathrm{Cov}(X_i^2+Y_i^2, Z_X^2+Z_Y^2) \\ &= \mathrm{Cov}(X_i^2,Z_X^2) + \mathrm{Cov}(Y_i^2,Z_Y^2) + \mathrm{Cov}(X_i^2,Z_Y^2) + \mathrm{Cov}(Y_i^2,Z_X^2) \end{aligned}$$

Let us start by examining the first term of $\mathrm{Cov}(R_i,P)$

$$\begin{aligned} \mathrm{Cov}(X_i^2,Z_X^2) &= \langle \left(X_i^2 - \langle X_i^2\rangle\right)\left(Z_X^2 - \langle Z_X^2\rangle\right)\rangle \\ &= \langle X_i^2 Z_X^2\rangle + \langle X_i^2\rangle\langle Z_X^2\rangle - \langle X_i^2\langle Z_X^2\rangle\rangle - \langle\langle X_i^2\rangle Z_X^2\rangle \\ &= \langle X_i^2 Z_X^2\rangle - \langle X_i^2\rangle\langle Z_X^2\rangle \end{aligned}$$

We have

$$\begin{cases} \langle X_i^2\rangle = \mathrm{Var}(X_i) + \langle X_i\rangle^2 = \sigma^2 \\ \langle Z_X^2\rangle = \mathrm{Var}(Z_X) + \langle Z_X\rangle^2 = n\sigma^2 \end{cases} \Rightarrow \langle X_i^2\rangle\langle Z_X^2\rangle = n\sigma^4.$$

If $i \in J$ we split Z_X in two parts to calculate $\langle X_i^2 Z_X^2\rangle$

$$Z_X = X_i + \sum_{j\in K} X_j \triangleq X_i + W_i$$

with $K = \{j \in J | j \neq i\}$. Since X_i and X_j are independent random variables for $i \neq j$, it follows that X_i is independent from W_i.

Note that $\langle W_i \rangle = 0$ and $\mathrm{Var}(W_i) = (n-1)\sigma^2$, resulting in

$$\begin{aligned}\langle X_i^2 Z_X^2 \rangle &= \langle X_i^2 (X_i + W_i)^2 \rangle \\ &= \langle X_i^2 (X_i^2 + W_i^2 + 2X_i W_i) \rangle \\ &= \langle X_i^4 \rangle + \langle X_i^2 W_i^2 \rangle + 2\langle X_i^3 W_i \rangle \\ &= 3\sigma^4 + \langle X_i^2 \rangle \langle W_i^2 \rangle + 2\langle X_i^3 \rangle \langle W_i \rangle \\ &= 3\sigma^4 + \sigma^2 (n-1)\sigma^2 \\ &= (2+n)\sigma^4\end{aligned}$$

and further leading to

$$\mathrm{Cov}(X_i^2, Z_X^2) = (2+n)\sigma^4 - n\sigma^4 = 2\sigma^4$$

Analagously, we find the second term of $\mathrm{Cov}(R_i, P)$ to be $\mathrm{Cov}(Y_i^2, Z_Y^2) = 2\sigma^4$. Since X_i is independent from Y_j for all j, and is thus independent from Z_Y, we find the third term of $\mathrm{Cov}(R_i, P)$ to be

$$\begin{aligned}\mathrm{Cov}(X_i^2, Z_Y^2) &= \langle \left(X_i^2 - \langle X_i^2 \rangle\right) \left(Z_Y^2 - \langle Z_Y^2 \rangle\right) \rangle \\ &= \langle X_i^2 - \langle X_i^2 \rangle \rangle \langle Z_Y^2 - \langle Z_Y^2 \rangle \rangle \\ &= 0\end{aligned}$$

Analagously we have $\mathrm{Cov}(Y_i, Z_X) = 0$, resulting in $\mathrm{Cov}(R_i, P) = 4\sigma^2$. Since we know R_i and P to be fully developed speckle patterns, their variances equal their mean intensity squared

$$\begin{cases} \mathrm{Var}(R_i) = I_0^2 = (2\sigma^2)^2 = 4\sigma^4 \\ \mathrm{Var}(P) = (nI_0)^2 = (2n\sigma^2)^2 = 4n^2\sigma^4 \end{cases}$$

Thus, when comparing one of the reference patterns R_i with a pattern P generated by n coherent beams, one of which is the ith beam, we find

$$\mathrm{Corr}(R_i, P) = \frac{4\sigma^4}{\sqrt{(4\sigma^4)(4n^2\sigma^4)}} = \frac{1}{n}$$

Similarly, when $i \notin J$ we find $\mathrm{Corr}(R_i, P) = 0$

Speckle pattern cross-correlation confidence bound

Here we construct a confidence interval to decide with 95% probability wether a measured cross-correlation coefficient ρ belongs to a zero-mean distribution $\mu_\rho = 0$. For our purposes, a one-sided confidence interval is most suited. Since correlation coefficients are in general not normally distributed we use the Fisher transform z defined as

$$z = \mathrm{atanh}(\rho).$$

When testing the hypothesis $\mu_\rho = 0$, the corresponding Fisher variable z has a zero-mean normal distribution with variance σ_z^2. Thus we can easily construct the required confidence bound on z as

$$P(z \leq 1.645\sigma_z) = 95\%.$$

This means that we can accept the hypothesis $\mu_\rho = 0$ with 95% confidence when

$$\rho \leq \tanh(1.645\sigma_z).$$

For our purposes σ_z is calculated in function of the number of speckle spots, as described in previous sections.
